# Prospective Five-Point Serial Assessment of Radiological and Functional Outcome Dissociation After Locking Plate Fixation of Proximal Tibial Fractures

**DOI:** 10.7759/cureus.113407

**Published:** 2026-07-26

**Authors:** Ovais Mehboob, Rabi R Prasad, Amit M Hegde, Hitesh Garg, Manu Gautam, Himanshu Gupta, Anshul Tripathi, Ravi Shankar

**Affiliations:** 1 Orthopedic Surgery, Max Super Speciality Hospital, New Delhi, IND; 2 Orthopedics, Max Super Speciality Hospital, New Delhi, IND; 3 Orthopedics, Sports Injury Center, Vardhman Mahavir Medical College and Safdarjung Hospital, New Delhi, IND; 4 Orthopedic Surgery, Dharamshila Narayana Superspeciality Hospital, New Delhi, IND; 5 Orthopedics, Vardhman Mahavir Medical College and Safdarjung Hospital, New Delhi, IND

**Keywords:** bicolumnar plating, dual-plate fixation, functional outcome, modified rasmussen criteria, patient-reported outcomes, proximal tibia locking plate, radiological-functional dissociation, schatzker classification, surgical outcome audit, tibial plateau fracture

## Abstract

Introduction: Orthopedic surgeons have traditionally assessed the success of tibial plateau fixation by evaluating radiological restoration of articular congruity, as observed on the initial postoperative X-ray. However, patient-centered outcomes such as pain, ambulation, knee stability, and return to work progress more slowly and in an unpredictable manner. This study investigated whether optimal radiological results on postoperative day 1 correspond to comparable functional recovery at six months, utilizing five serial assessments of both radiological and patient-focused functional outcomes in a prospective cohort treated with fracture morphology-guided locking plate fixation.

Methods: A prospective observational study enrolled 32 patients with closed proximal tibial fractures (Schatzker types I-VI) over eight months (January-August 2023). Outcome assessment using the modified Rasmussen criteria was performed preoperatively and at the immediate postoperative, suture removal, three-month, and six-month intervals. Statistical analysis used the Friedman test with Wilcoxon signed-rank pairwise comparisons (p < 0.05).

Results: The mean patient age was 38.53 ± 11.20 years, with 53.1% (17/32) male participants. Schatzker type IV fractures were most common (31.3%, n = 10). Bicolumnar dual plating was utilized in six patients (18.8%). The mean modified Rasmussen clinical score rose to 23.88 ± 1.96 at six months, and the radiological score rose from 0.78 ± 0.91 preoperatively to 8.44 ± 0.91 (both p < 0.001). At six months, all 32 patients (100%) had reached a good or excellent radiological grade (excellent 40.6%, n = 13; good 59.4%, n = 19), whereas only 14 patients (43.8%) had reached a good or excellent clinical grade; 18 (56.3%) remained fair, and just one (3.1%) achieved an excellent clinical grade. Radiological and clinical scores at six months showed no correlation (Spearman ρ = 0.101, p = 0.581), and none of the 13 patients with an excellent radiograph achieved an excellent functional grade. No complications were recorded on protocol-defined surveillance.

Conclusion: Morphology-guided proximal tibial fixation reliably restores radiological parameters by six months. Functional recovery, however, lags markedly behind and is uncorrelated with radiological score, with most patients still in the fair clinical grade at six months. This radiological-functional dissociation is reported here as an early postoperative observation; because functional recovery after tibial plateau fracture commonly continues for 12-24 months, the gap documented at six months is expected to narrow with longer follow-up, and its resolution cannot be characterized by this dataset. These findings derive from a small single-center cohort and require confirmation in larger multicenter studies. Nonetheless, they indicate that surgeons should not equate a satisfactory postoperative radiograph with treatment success and should audit outcomes with patient-related functional measures over adequate follow-up.

## Introduction

Proximal tibial fractures constitute approximately 1% of all skeletal fractures and up to 8% of fractures in the elderly population, encompassing injuries ranging from low-energy simple splits to high-energy bicondylar fractures with extensive comminution and soft-tissue compromise [1,2]. The knee joint, as the fulcrum between the longest lever arms of the lower limb, requires near-anatomical articular restoration following intra-articular fracture to prevent premature post-traumatic osteoarthritis and long-term disability [3].

The Schatzker classification is the most widely utilized system for surgical planning, categorizing fractures from type I (lateral split) to type VI (metaphyseal-diaphyseal dissociation) [4]. Types I-III are primarily unicondylar lateral injuries suitable for single lateral plating, whereas types IV-VI involve medial column disruption, bicondylar depression, or metaphyseal extension, making single-plate constructs biomechanically insufficient. Cadaveric biomechanical studies have consistently shown that dual-plate fixation provides significantly greater rotational stability, reduced subsidence, and lower rates of varus collapse compared to unilateral constructs in bicondylar fracture models [5,6].

Current treatment strategies for complex tibial plateau fractures emphasize morphology-guided, column-specific fixation using periarticular locking plates, which offer fixed-angle stability in osteoporotic or comminuted bone [7]. The approach of bicolumnar or dual plating, which addresses both the medial and lateral tibial columns through separate incisions, has become the preferred treatment for Schatzker types V and VI, as well as selected type IV fractures [8,9]. The medial column, responsible for bearing approximately 60% of the axial knee load, is structurally essential. Inadequate independent support of the medial column results in progressive varus deformity and articular step-off, even when the lateral column appears sufficiently stabilized [10].

Although evidence supporting bicolumnar plating continues to grow, a fundamental concern persists in the surgical literature on tibial plateau fractures: are the most relevant outcomes being measured? The orthopedic community has traditionally equated a satisfactory postoperative radiograph with surgical success. While articular congruity, mechanical axis restoration, and implant positioning are reliably achievable and easily measured, these parameters reflect surgical technique rather than patient recovery. Functional recovery, including pain relief, return to occupation, restoration of knee stability, and gait, depends on soft-tissue healing, quadriceps rehabilitation, proprioceptive retraining, and psychosocial factors that cannot be addressed by implants alone. Continued reliance on radiological endpoints as primary success metrics risks overlooking patients who appear well on radiographs at six weeks but remain functionally impaired at six months or longer. This study was designed to prospectively document this difference by tracking both radiological and patient-focused clinical outcomes at five serial time points in a cohort managed with fracture morphology-guided locking plate fixation. The central argument is that radiological perfection is a prerequisite but not the endpoint of successful tibial plateau surgery, and that outcomes should be audited using patient-related functional measures over an adequate follow-up period.

## Materials and methods

Study design and setting

This single-center, prospective observational study was conducted in the Department of Orthopedics at Max Super Specialty Hospital, Shalimar Bagh, New Delhi, between January 2023 and August 2023. Institutional ethical approval was secured before participant recruitment, and written informed consent was obtained from all participants in their preferred language. The study adhered to the principles outlined in the Declaration of Helsinki.

Sample size and participants

**Figure 1:**
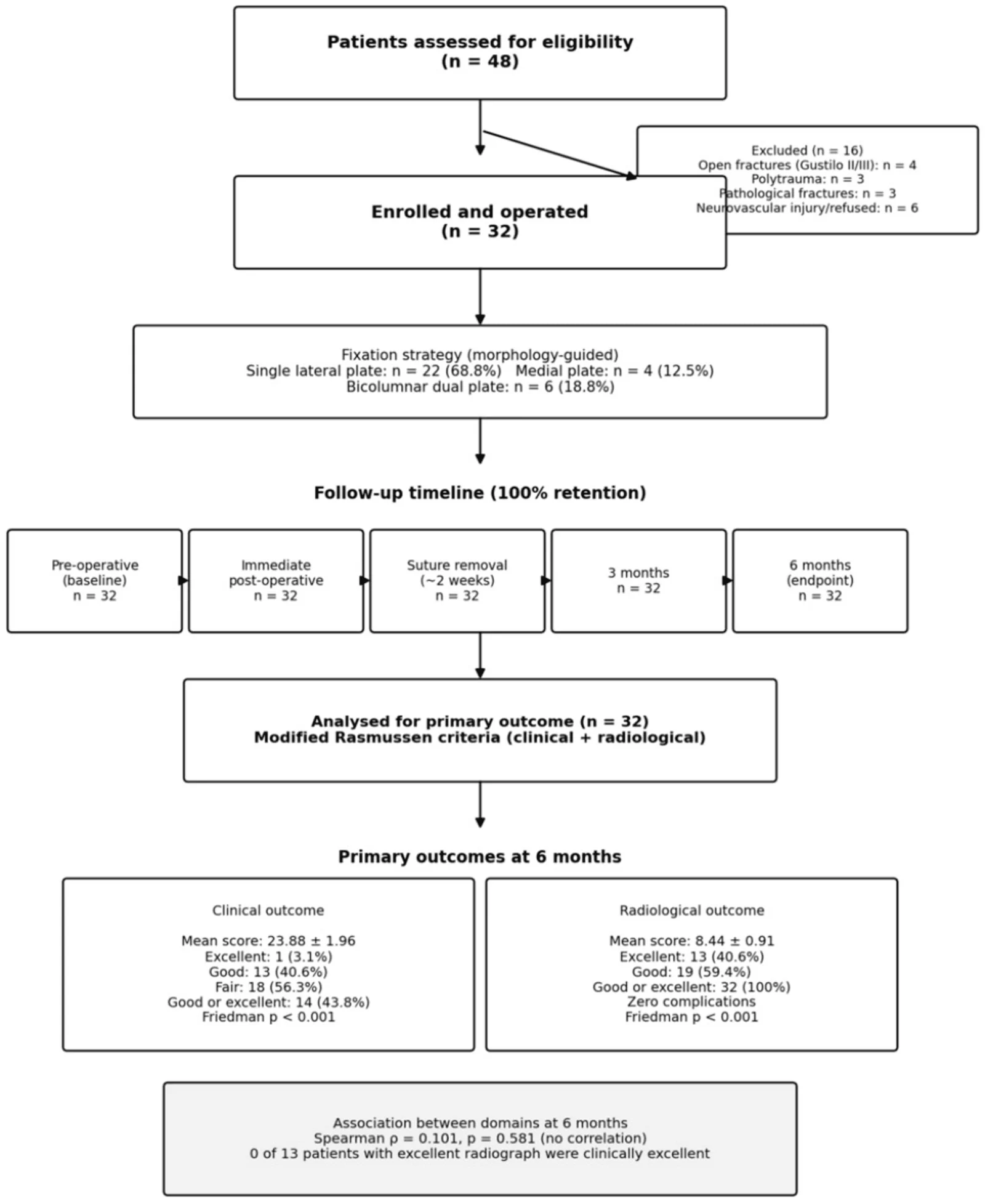
STROBE flow diagram illustrating patient recruitment, exclusion, allocation by fixation strategy, follow-up timeline, and primary outcomes STROBE, Strengthening the Reporting of OBservational studies in Epidemiology

Based on an assumed prevalence of tibial plateau fractures requiring surgical fixation of 1%, with a 3.5% margin of error at a 5% significance level (Z = 1.96), the minimum calculated sample size was 32 patients. Participants were recruited sequentially. Inclusion criteria included age of 18 years or older, closed tibial plateau fracture (Schatzker types I-VI), and provision of written informed consent. Exclusion criteria comprised open fractures, polytrauma, pathological fractures, associated neurovascular injury, and medical unfitness for surgery. The flow of patients through recruitment, allocation, and follow-up is depicted through the Strengthening the Reporting of OBservational studies in Epidemiology diagram shown in Figure 1 [11].

Operative technique

Surgical procedures were performed under spinal or general anesthesia on a radiolucent operating table with tourniquet control. The fixation strategy was determined by fracture pattern: Schatzker types I-III were treated with single anterolateral plating; type III fractures with significant depression also received subchondral bone grafting; type IV fractures were managed with medial buttress or posteromedial plating; and types V and VI were treated with bicolumnar dual plating through two separate incisions.

**Figure 2:**
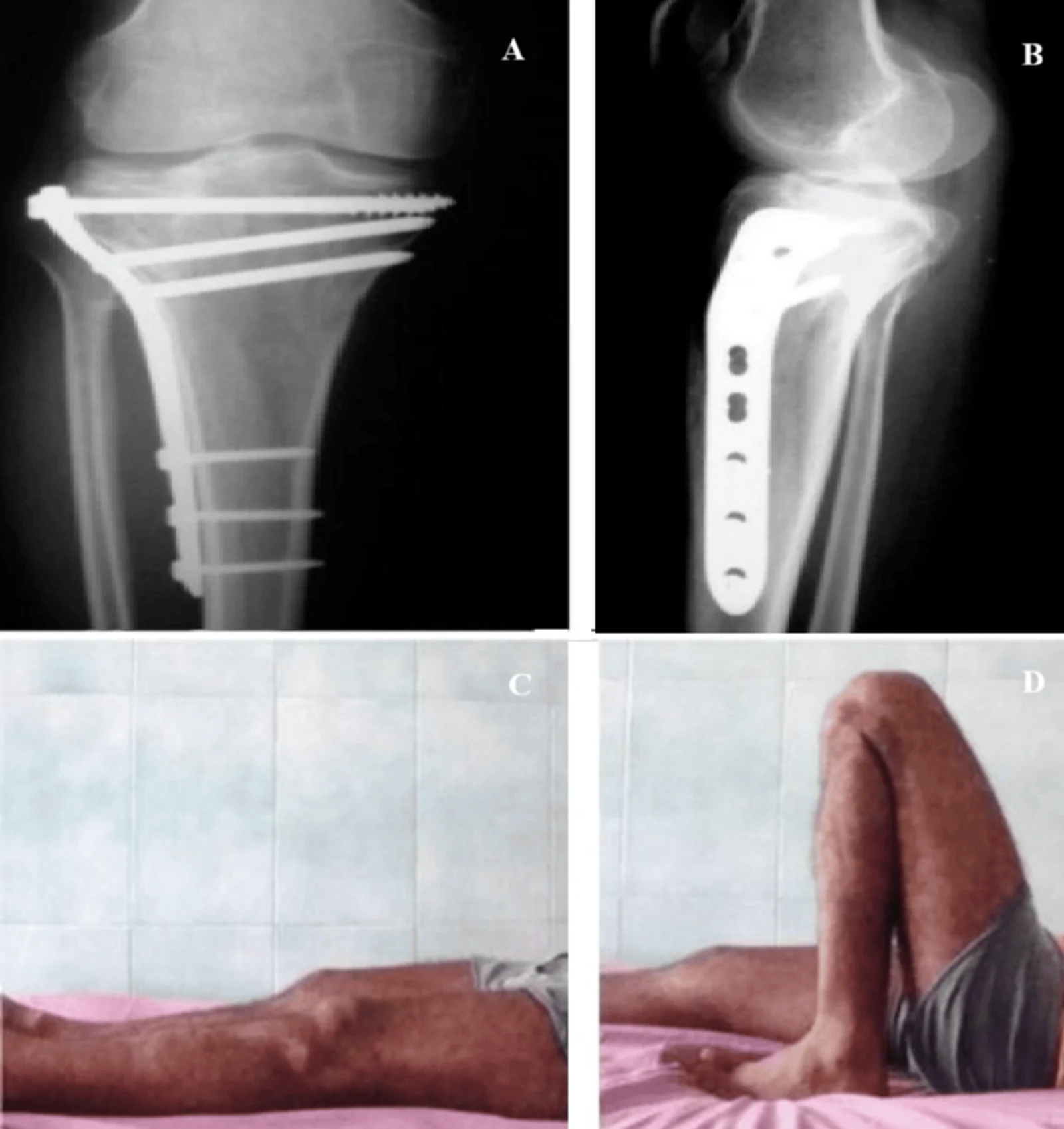
Bone grafting done for the depressed proximal tibia fracture Postoperative (A) anteroposterior and (B) lateral radiographs demonstrating bone grafting and fixation of a depressed proximal tibial fracture. (C,D) Clinical photographs demonstrating postoperative knee range of motion

For bicolumnar fixation, the medial column was addressed first using either a 2-4 cm minimally invasive medial incision or a formal posteromedial approach (interval between the pes anserinus and medial gastrocnemius head), with a 3.5 mm proximal medial tibial locking plate or T-buttress plate. The lateral column was subsequently fixed through an anterolateral approach (Gerdy’s tubercle curvilinear incision) with an anatomical lateral locking plate. Articular reduction was confirmed fluoroscopically prior to definitive fixation. Metaphyseal voids were filled with autologous iliac crest graft or synthetic bone substitute in 50% of cases, as shown in Figure 2.

Rehabilitation protocol

Static quadriceps exercises and gentle knee mobilization began on postoperative day 1. Non-weight-bearing ambulation was maintained for six to eight weeks, followed by progression to partial weight-bearing at 8-12 weeks, and full weight-bearing at 12-16 weeks, dependent on radiological evidence of union (cortical continuity in at least three cortices on anteroposterior and lateral projections).

Outcome assessment

The primary outcome was the modified Rasmussen score [12], assessed at five time points: preoperative baseline, immediate postoperative (prior to discharge), suture removal (approximately two weeks), three months, and six months. The clinical subscale (maximum 30 points) evaluates pain, walking capacity, knee extension, range of motion (Figure 2), stability, and quadriceps power. The radiological subscale (maximum 10 points) evaluates articular depression, condylar widening, varus or valgus angulation, and progression of osteoarthrosis. Grades were classified as excellent (clinical ≥28; radiological 9-10), good (24-27; 7-8), fair (20-23; 5-6), and poor (<20; <5).

Statistical analysis

The nonparametric Friedman test was used for repeated-measures comparison of Rasmussen scores across time points. Pairwise comparisons were performed using the Wilcoxon signed-rank test with Bonferroni correction. For the radiological score, all five assessment points (preoperative through six months) were included. For the clinical score, a data-verification pass against the source registry identified that preoperative clinical subscale totals were not interpretable on the 0-30 scale; the preoperative clinical value was therefore excluded from analysis altogether, and the repeated-measures comparison was restricted to the four verified postoperative assessment points (immediate postoperative through six months). The association between the clinical and radiological domains at six months was assessed using the Spearman rank correlation coefficient, and grade concordance between domains by cross-tabulation. Between-group comparisons were performed using the independent-samples t-test (continuous variables) or chi-square test (categorical variables). Statistical significance was defined as p < 0.05. All analyses were carried out using the Statistical Package for the Social Sciences, version 26.0 (IBM Corp., Armonk, NY).

## Results

Patient demographics

Thirty-two patients were recruited with 100% follow-up at all five time points. Table 1 presents demographic and perioperative characteristics. The mean age was 38.53 ± 11.20 years (range 22-65); 17 (53.1%) were male and 15 (46.9%) female patients, with no significant sex difference in age (p = 0.782). The majority (81.3%) were engaged in heavy-load occupations, and 75% presented within seven days of injury.

**Table 1:** Patient demographics and perioperative characteristics Continuous variables compared by independent-samples t-test (age and blood loss: t statistic reported; operative time: t statistic not available from archived source data); categorical variables (occupation, injury-to-surgery interval) compared by chi-square test (χ² statistic reported) SD, standard deviation; ns, not significant

Parameter	Female (n = 15)	Male (n = 17)	Total (n = 32)	p value
Age (years), mean ± SD	37.93 ± 11.11	39.06 ± 11.60	38.53 ± 11.20	0.782 (t = 0.28, df = 30, ns)
Age range (years)	22-65	24-62	22-65	-
Heavy-load occupation	12 (80.0%)	14 (82.4%)	26 (81.3%)	0.861 (χ² = 0.03, df = 1, ns)
Injury to surgery ≤7 days	11 (73.3%)	13 (76.5%)	24 (75.0%)	0.823 (χ² = 0.04, df = 1, ns)
Mean blood loss (mL)	396.4 ± 31.2	405.3 ± 37.1	401.2 ± 34.4	0.471 (t = 0.73, df = 30, ns)
Mean operative time (minutes)	98.6 ± 22.4	101.2 ± 19.8	99.9 ± 20.9	0.685 (ns)

Fracture distribution and fixation strategy

Schatzker type IV was the most prevalent fracture pattern (31.3%), followed by type II (25.0%), types V and VI (12.5% each), and types I and III (9.4% each). Single lateral plating was performed in 22 patients (68.8%), bicolumnar dual plating in 6 (18.8%), and medial plating alone in 4 (12.5%). Bone grafting was required in 16 patients (50%). The quality of reduction was satisfactory or better in all patients. Table 2 details fracture distribution against treatment modality.

**Table 2:** Fracture distribution and treatment modality

Schatzker type	n (%)	Dual plate	Lateral plate	Medial plate	Bone graft used
Type I (lateral split)	3 (9.4%)	0	3 (100%)	0	0
Type II (split-depression)	8 (25.0%)	0	8 (100%)	0	5 (62.5%)
Type III (pure depression)	3 (9.4%)	0	3 (100%)	0	3 (100%)
Type IV (medial plateau)	10 (31.3%)	3 (30%)	3 (30%)	4 (40%)	4 (40%)
Type V (bicondylar)	4 (12.5%)	1 (25%)	3 (75%)	0	2 (50%)
Type VI (metaphyseal dissociation)	4 (12.5%)	2 (50%)	2 (50%)	0	2 (50%)
Total	32 (100%)	6 (18.8%)	22 (68.8%)	4 (12.5%)	16 (50%)

Clinical functional outcome

**Figure 3:**
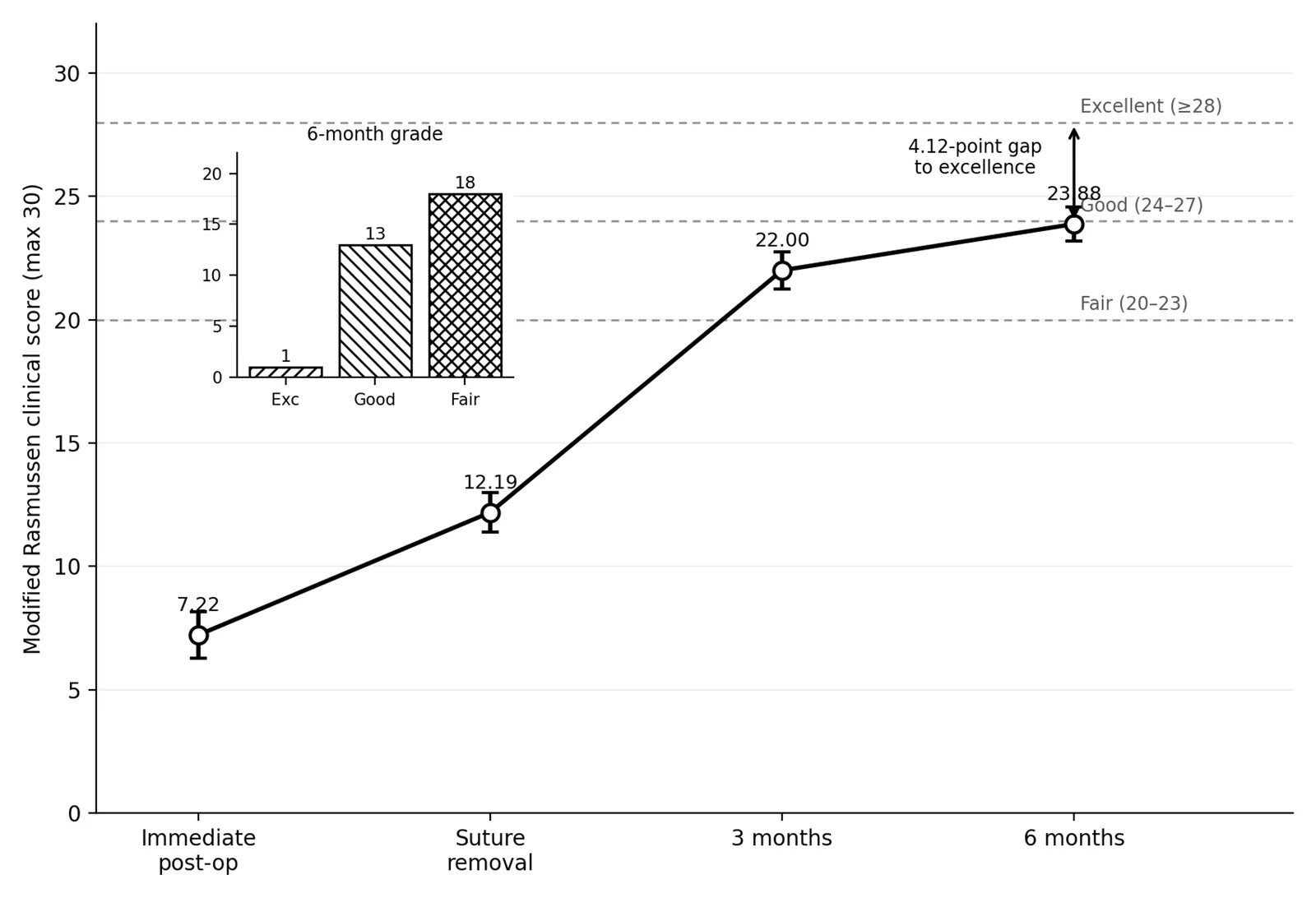
Serial modified Rasmussen clinical score trajectory at five time points (n = 32). Error bars represent 95% confidence intervals. Inset shows six-month grade distribution. All pairwise comparisons are significant (Wilcoxon signed-rank, p < 0.001) Serial modified Rasmussen clinical score trajectory across the four verified postoperative time points (n = 32). Error bars represent 95% confidence intervals. Gray dashed lines mark the clinical grade thresholds (Excellent ≥28; Good 24-27; Fair 20-23). The arrow highlights the 4.12-point gap between the six-month mean (23.88) and the excellence threshold (28), corresponding to the 18 patients (56.3%) still graded fair at that time point. The inset shows the six-month grade distribution. All pairwise comparisons were significant (Wilcoxon signed-rank, p < 0.001; test statistics are reported in Table 3) Exc, excellent

Table 3 presents serial modified Rasmussen clinical scores. The mean score improved monotonically across all verified intervals, from 7.22 ± 2.72 immediately postoperatively to 23.88 ± 1.96 at six months (Friedman test across the four verified postoperative assessment points: χ² = 88.75, df = 3, p < 0.001), with all pairwise Wilcoxon comparisons against the immediate postoperative time point statistically significant (p < 0.001). The preoperative clinical value was excluded from analysis (see the Methods section). At six months, one patient (3.1%) achieved an excellent clinical grade, 13 (40.6%) good, and 18 (56.3%) fair; no patient remained in the poor category. Fourteen patients (43.8%) had, therefore, reached a good or excellent clinical grade. The serial progression is illustrated in Figure 3. The clinical recovery curve was still ascending at six months, indicating that functional improvement was ongoing at the end of the study window and had not plateaued.

**Table 3:** Serial modified Rasmussen clinical scores at five time points Friedman test across the four verified postoperative assessment points (χ² = 88.75, df = 3, p < 0.001) with Wilcoxon signed-rank pairwise post hoc comparisons vs. immediate postoperative (Suture removal: Z = -4.78; 3 months: Z = -4.94; 6 months: Z = -4.94; all p < 0.001, Bonferroni-corrected α = 0.0167). The preoperative clinical value was excluded from analysis following source-data verification (see the Methods section) ^*^p < 0.001 Exc, excellent; SD, standard deviation; CI, confidence interval

Time point	Mean ± SD	95% CI (lower)	95% CI (upper)	Grade, n (%)	p value vs. post-op
Preoperative	Not analyzed	-	-	-	-
Immediate post-op	7.22 ± 2.72	6.28	8.16	Poor: 32 (100%)	Reference
Suture removal (~2 weeks)	12.19 ± 2.32	11.38	12.99	Poor: 32 (100%)	<0.001^*^
3 months	22.00 ± 2.16	21.25	22.75	Good: 8 (25.0%); Fair: 22 (68.8%); Poor: 2 (6.2%)	<0.001^*^
6 months	23.88 ± 1.96	23.19	24.56	Exc: 1 (3.1%); Good: 13 (40.6%); Fair: 18 (56.2%)	<0.001^*^

Radiological outcome

Table 4 presents serial modified Rasmussen radiological scores. The mean score improved from 0.78 ± 0.91 preoperatively to 8.44 ± 0.91 at six months (Friedman p < 0.001). All 32 patients (100%) achieved an excellent radiological grade by six months, a complete transition from 100% poor at baseline. No secondary loss of reduction or secondary malalignment was observed at any subsequent interval. In direct contrast to the clinical functional results, where 56.3% of patients remained in the fair grade at the same time point, radiological recovery was universal and early. This difference between the two outcome domains is the central finding of the study and is discussed in detail below.

**Table 4:** Serial modified Rasmussen radiological scores at five time points Friedman test (χ² = 124.17, df = 4, p < 0.001) with Wilcoxon signed-rank pairwise post hoc comparisons vs. preoperative (immediate post-op: Z = -4.69; Suture removal: Z = -4.90; 3 months: Z = -4.97; 6 months: Z = -4.98; all p < 0.001, Bonferroni-corrected α = 0.0125) ^*^p < 0.001 Exc, excellent; SD, standard deviation; CI, confidence interval

Time point	Mean ± SD	95% CI (lower)	95% CI (upper)	Grade, n (%)	p value vs. pre-op
Preoperative	0.78 ± 0.91	0.45	1.11	Poor: 32 (100%)	Reference
Immediate post-op	2.44 ± 0.72	2.18	2.70	Poor: 32 (100%)	<0.001^*^
Suture removal (~2 weeks)	4.06 ± 1.01	3.70	4.43	Good: 10 (31.3%); Fair: 22 (68.7%)	<0.001^*^
3 months	5.50 ± 0.88	5.18	5.82	Exc: 3 (9.3%); Good: 25 (78.1%); Fair: 4 (12.5%)	<0.001^*^
6 months	8.44 ± 0.91	8.11	8.77	Excellent: 32 (100%)	<0.001^*^

Perioperative parameters and complications

**Figure 4:**
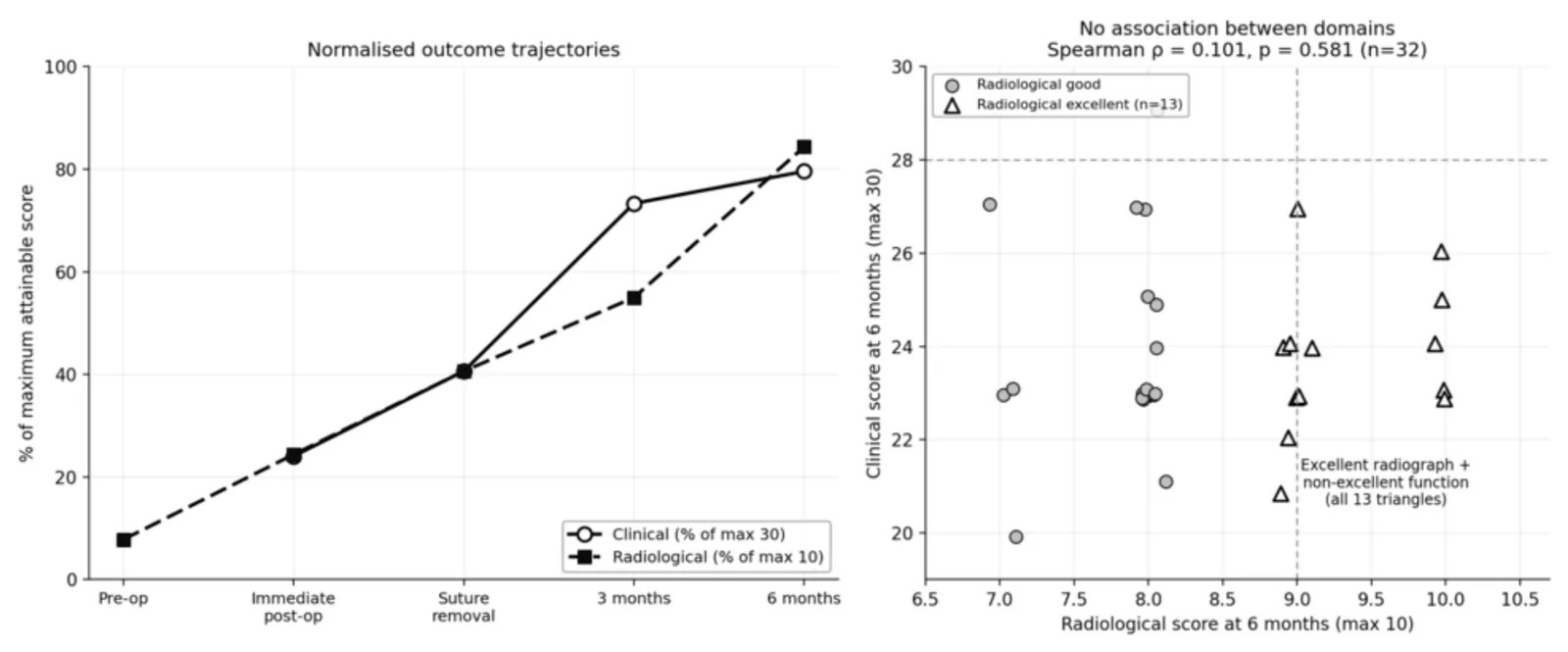
Comparative clinical and radiological outcome trajectories (normalized to the percentage of maximum score) at five time points. Right: six-month grade distributions and key performance indicators. Left: the clinical and radiological scores as a percentage of their respective maxima across all assessment points; the two curves track closely until suture removal and then separate, the radiological curve rising more steeply to six months. Right: each patient's six-month clinical score against their radiological score. Points are widely scattered with no discernible trend, consistent with the absence of correlation (Spearman ρ = 0.101, p = 0.581)

Table 5 summarizes intraoperative parameters stratified by fixation type. The comparative clinical and radiological trajectories, normalized to the percentage of the maximum score, are displayed in Figure 4. Bicolumnar constructs were associated with higher mean blood loss (452 vs. 386 mL) and longer operative duration (138 vs. 92 minutes), consistent with the additional medial exposure. However, no wound complications, deep infections, compartment syndrome, implant failures, malunions, or nonunions were recorded. Mean time to radiological union was 15.2 ± 2.0 weeks.

**Table 5:** Intraoperative parameters and complication profile by fixation type

Parameter	Dual plate (n = 6)	Single plate (n = 26)	Overall (n = 32)
Mean blood loss, mL (range)	452 (380-530)	386 (347-457)	401 ± 34.4
Mean operative time, minutes	138 (120-160)	92 (60-140)	99.9 ± 20.9
Bone graft required, n (%)	3 (50%)	13 (50%)	16 (50%)
Quality of reduction			
Excellent	2 (33.3%)	0 (0%)	2 (6.3%)
Satisfactory	4 (66.7%)	22 (100%)	30 (93.8%)
Postoperative complications	0 (0%)	0 (0%)	0 (0%)
Radiological union (mean weeks)	16.2 ± 2.1	14.8 ± 1.9	15.2 ± 2.0
Radiation exposure (<20 dGy/cm²)	6 (100%)	26 (100%)	32 (100%)

Figure 4 displays the two outcome domains together. The 13 patients who reached an excellent radiological grade (open triangles, right of the vertical reference line) are distributed across the good and fair clinical bands, and all fall below the excellent clinical threshold (horizontal reference line), illustrating that a structurally excellent radiograph did not correspond to excellent function in any patient.

## Discussion

In clinical practice, orthopedic surgeons frequently gauge surgical success by reviewing postoperative radiographs. In tibial plateau surgery, this tendency is perpetuated by the design of most published outcome series, which focus on radiological grading, such as articular step-off, condylar widening, and mechanical axis, while treating functional scores as secondary metrics. The present study challenges this hierarchy. At six months, every patient had achieved a good or excellent Rasmussen radiological grade; however, only 43.8% reached a good or excellent functional clinical grade, 56.3% remained fair, and just one patient (3.1%) was clinically excellent. Crucially, the two domains were not merely divergent in aggregate; they were statistically unrelated at the level of the individual patient (Spearman ρ = 0.101, p = 0.581), and not one of the 13 patients with an excellent radiograph achieved an excellent functional grade. This dissociation is not exclusively attributable to short follow-up duration. Rather, it reflects an underlying biological reality: bone healing occurs more rapidly than recovery of neuromuscular, proprioceptive, and soft-tissue systems that govern functional outcomes. Quadriceps inhibition following intra-articular knee trauma can persist for months, independent of fracture union [3]. Proprioceptive deficits after tibial plateau fracture have been documented even at 12 months [13]. The recovery curves for radiological and functional parameters follow fundamentally different trajectories, and conflating them risks an overly optimistic assessment of surgical success.

These observations do not diminish the importance of anatomical reduction and stable fixation; rather, these are essential prerequisites. In complex bicondylar injuries, bicolumnar plating creates the mechanical conditions required for functional recovery. The medial tibial condyle supports approximately 60% of axial knee load [10]; inadequate buttressing results in progressive varus collapse, articular step-off, and ultimately post-traumatic arthritis, which precludes functional recovery. Cadaveric biomechanical studies have demonstrated that dual-plate constructs provide greater rotational stability, reduced subsidence, and lower rates of varus collapse compared to unilateral fixation in bicondylar fracture models [5,6]. Raj et al. reported that all fractures in their dual plating series united without varus collapse, with 76.7% achieving excellent or good Rasmussen functional scores at 24 months [14], a result that necessitates both adequate fixation and sufficient follow-up duration. Senthurvelan et al. reported a mean final Rasmussen functional score of 25.48 in their bicolumnar series at last follow-up [8], and Vasiliadis et al. documented good-to-excellent mid-term functional outcomes at seven years [15]. These longer term datasets indicate that meaningful functional improvement demands a recovery period that six months cannot provide, even when early radiological outcomes are optimal.

In the present series, all six bicolumnar patients achieved a good or excellent radiological grade by six months (mean 8.50 ± 1.22), with no secondary loss of reduction, supporting construct adequacy. These radiological outcomes are consistent with findings by Caglar et al., who reported superior condylar width maintenance and restoration of the medial proximal tibial angle with dual locked plating compared with single lateral plating for comminuted fractures [16]. However, radiological metrics alone do not provide information regarding a patient's ability to return to work, ambulate without a limp, or ascend stairs without pain. Such functional outcomes can only be assessed through validated functional scores, which require follow-up intervals sufficient for recovery to plateau.

The radiological-functional dissociation: understanding the gap

The principal finding of this study is not the expected uniformity of radiological recovery; every patient reached a good or excellent grade by six months, but that only one patient (3.1%) achieved an excellent functional clinical grade at the same time point, while 18 (56.3%) remained fair, and that the two domains showed no correlation whatsoever (Spearman ρ = 0.101, p = 0.581). This outcome demonstrates the radiological-functional dissociation within a prospective serial dataset and does so at the level of the individual patient rather than by comparison of group means. This phenomenon does not represent a complication; it is a biological reality that has not been adequately emphasized in the orthopedic literature. A retrospective analysis using the same modified Rasmussen instrument similarly found categorical agreement between clinical-functional and radiological grades in only 34% of tibial plateau fracture patients at a minimum 18-month follow-up [17], supporting the dissociation observed here; the present series extends this observation by demonstrating that the gap is already detectable within the first six months, while radiological healing is still in progress and functional recovery has not yet plateaued. The modified Rasmussen clinical score at six months (23.88 ± 1.96) represents 79.6% of the maximum possible score, a mean that appears creditable until it is noted that the threshold for excellence begins at 28, and 56.3% of patients have not even reached the good band. These patients do not represent failures of fixation, as their radiographs are optimal. Rather, their muscles, ligaments, proprioceptors, and confidence in the repaired limb have not yet matched the structural repair. The stepwise improvement in clinical scores, statistically significant at every interval, indicates that recovery was ongoing at the end of the study period. Extending follow-up to 12 and 24 months, as demonstrated by Raj et al. [14] and Vasiliadis et al. [15], consistently reveals migration of functional grades from fair to good and excellent, which is not observable at six months. Maniar and Patel reported a mean Rasmussen functional score of 24.55 ± 3.17 at six months [18], consistent with a good-predominant distribution, while Raj et al. found 76.7% excellent or good at 24 months [14]. The trajectory of recovery carries as much clinical significance as any single time-point measurement. Surgeons who confine their assessment to the six-week radiograph, or who favor radiological metrics over patient-centered outcomes, are unlikely to observe this progression.

Radiological outcome data should be interpreted within this context. The mean Rasmussen radiological score reached 8.44 ± 0.91 at six months, with 40.6% of patients graded excellent and the remaining 59.4% good, no patient fair or poor, representing a complete transition from 100% poor at baseline. No secondary loss of reduction or malalignment was observed. These findings attest to the technical success of the operative strategy. However, they also demonstrate why reliance on radiological endpoints can lead to premature conclusions regarding overall success: universal good-to-excellent radiological outcomes contrasted with only 43.8% good-to-excellent clinical function at the same time point in the same cohort, with no correlation between the two. This variation, as documented in this prospective dataset, signals the necessity of supplementing radiological metrics with patient-related functional outcome measures. The absence of complications further supports the adequacy of the surgical technique and excludes surgical error as a cause for the observed functional lag, thereby underscoring the roles of structured rehabilitation and time in ongoing recovery.

Auditing surgical success through patient-related functional outcomes

The orthopedic community has long faced practical challenges in adopting patient-reported outcome measures (PROMs). These instruments are time-intensive to administer, subject to cultural variability, less amenable to concise summary statistics, and require extended follow-up periods that can be operationally difficult in high-volume trauma centers. In contrast, radiographs are rapid and objective, and deliver clear documentation of anatomical repair. However, radiographs do not address the patient's primary concerns, such as the ability to return to work, ambulate independently, or experience pain relief. Shifting from radiological to functional evaluation of surgical outcomes is not merely a methodological choice but an ethical obligation to maximize patient recovery. Validated, patient-administered instruments such as the Knee injury and Osteoarthritis Outcome Score (KOOS), Western Ontario and McMaster Universities Osteoarthritis Index (WOMAC), International Knee Documentation Committee, and Lysholm scores capture aspects of recovery that radiographs cannot assess [19-22]. The present study used the modified Rasmussen criteria as a combined instrument; its clinical subscale is surgeon-assessed rather than patient-reported, which constitutes a limitation. It is worth noting that this limitation is unlikely to have manufactured the dissociation reported here: a surgeon-assessed instrument, if anything, tends toward optimism relative to a patient-completed one, so a true patient-reported measure would plausibly have shown an equal or wider gap. Nevertheless, the serial five-point trajectory documented here clearly illustrates the functional lag that PROMs would likely capture with even greater sensitivity. The observation that clinical recovery was ongoing at the six-month endpoint gives essential information for patient counseling, which would be missed if outcome assessment concluded at the six-week radiograph. Surgeons managing proximal tibial fractures should implement prospective outcome registries using validated PROMs, with a minimum of 12 months and ideally 24 months of follow-up, as the standard for audit. Radiological grading should serve as a quality-control measure for fixation constructs, but not as a surrogate for patient functional outcomes. The present dataset empirically substantiates this distinction: radiological outcomes were optimal, yet patients continued to recover functionally.

The existing comparative literature supports this perspective. Raj et al. found no significant difference in the Oxford Knee Score or the Rasmussen functional score between single- and dual-plate groups at final follow-up [23], a result evident only at 24 months and undetectable at a six-week radiological review. Çağlar et al. reported superior mechanical axis parameters with dual plating for Arbeitsgemeinschaft für Osteosynthesefragen/Orthopaedic Trauma Association 41-C3 fractures [16], but their functional outcome data also required extended follow-up to achieve statistical significance. A consistent pattern emerging from the literature is that radiological differences between implant strategies appear early and are readily measured, whereas functional differences, which are of primary importance to patients and clinical systems, develop gradually, require validated instruments for assessment, and necessitate follow-up durations that many single-center case series do not provide. The implication for surgical audit is clear: datasets lacking PROMs at 12 months or beyond cannot adequately address the most clinically relevant questions.

Technical progress in tibial plateau surgery, including minimally invasive posteromedial plating, variable-angle locking constructs, and refined soft-tissue staging protocols, has reduced perioperative morbidity and improved the reproducibility of radiological outcomes. However, the infrastructure for measuring subsequent functional recovery has lagged behind. Achieving excellent functional outcomes following optimal radiological results requires structured rehabilitation, active patient participation, and systematic functional assessment at defined intervals. In the present series, static quadriceps exercises and early mobilization began on day 1, with full weight-bearing delayed until radiological union, reflecting current best practice. Despite this, 56.3% of patients at six months remained in the fair category for functional grading, suggesting that muscular and proprioceptive recovery trailed behind the structural repair achieved by the implants. The biomechanical success of the fixation strategy, evidenced by zero loss of reduction, zero varus collapse, and 100% radiological union at a mean of 13.5 weeks, laid the groundwork for functional recovery but did not guarantee its realization and did not predict it. This distinction encapsulates the central message of this series.

Limitations

This study has several limitations that must be acknowledged. The sample size (n = 32) and follow-up duration of six months are the major constraints: they capture the early phase of recovery but not the functional plateau that typically emerges beyond 12 months. The radiological-functional dissociation documented here is therefore an early postoperative observation, not a demonstration of a permanent phenomenon; it is likely to narrow with time, and this series cannot characterize that evolution. The single-center design and absence of a randomized control group preclude causal inference, and the findings require confirmation in larger multicenter cohorts. The cohort spans Schatzker types I-VI managed by three different fixation strategies; although the dissociation was present within every fixation and severity stratum, the bicolumnar (n = 6) and medial-plate (n = 4) subgroups are underpowered for formal comparison or for multivariable adjustment by fracture type, and stratified results are reported descriptively only. Baseline data were confined to those variables captured by the study proforma; body mass index, smoking status, comorbidity burden, and associated ligamentous or meniscal injury were not systematically recorded and could not be retrieved retrospectively, so residual confounding by these factors cannot be excluded. Adherence to the rehabilitation protocol was supervised, but not quantified, and return-to-work status was not formally captured, an omission of particular consequence given that 81.3% of this cohort held heavy-load occupations. Preoperative clinical subscale totals in the source registry were not interpretable on the 0-30 scale and were excluded from analysis; therefore, the magnitude of change from baseline in the clinical domain cannot be quantified. The preoperative radiological score was unaffected. The primary outcome instrument, modified Rasmussen criteria, is surgeon-assessed rather than patient-reported; while widely used and validated for comparison with the existing literature, it does not completely capture patient-perceived pain, quality of life, or return to occupational and recreational function. The absence of patient-reported outcome instruments (KOOS, WOMAC, Lysholm, EuroQol 5-Dimension) represents the most important methodological gap for future work. Computed tomography (CT)-based three-column classification [24] was not systematically employed, and bone graft type was not standardized across cases. Future prospective multicenter studies with validated PROMs, a minimum two-year follow-up, and CT-based fracture characterization are required to fully characterize the functional recovery trajectory and the duration of the radiological-functional dissociation documented here.

## Conclusions

Fracture morphology-guided locking plate fixation for proximal tibial fractures, including bicolumnar constructs for complex bicondylar injuries, reliably achieves good-to-excellent radiological outcomes at six months with a zero complication rate on protocol-defined surveillance. However, the central finding of this prospective series is that radiological success and functional recovery are dissociated outcomes operating on different biological timescales. While 100% (32/32) of patients achieved a good or excellent radiological grade by six months, only 43.8% (14/32) achieved a good or excellent clinical functional grade at the same time point, 56.3% remained fair, and the two domains were entirely uncorrelated (Spearman ρ = 0.101, p = 0.581); none of the 13 patients with an excellent radiograph was functionally excellent. This dissociation was not a complication of surgery; it is an expected feature of recovery from complex periarticular trauma that the surgical literature has been slow to foreground. It must, however, be interpreted as an early postoperative observation: functional recovery after tibial plateau fracture commonly continues for 12-24 months, and this six-month window cannot establish whether, or when, the gap closes. These conclusions rest on a small, single-center cohort assessed with a surgeon-rated instrument and require confirmation in larger multicenter studies using validated patient-reported measures with extended follow-up. Surgeons managing tibial plateau fractures must resist the temptation to equate a satisfactory postoperative image with a successful outcome. Radiological perfection is the necessary foundation; it is not the measure of what the patient has recovered. Systematic audit of surgical outcomes must be performed using validated patient-related functional outcome instruments at adequate follow-up intervals, with a minimum of 12 months and ideally 24 months or beyond. The X-ray confirms what was fixed. The patient’s functional score tells you whether they were truly treated.
